# rs3806265 and rs4612666 of the *NLRP3* Gene Are Associated With the Titer of Glutamic Acid Decarboxylase Antibody in Type 1 Diabetes

**DOI:** 10.3389/fendo.2022.835054

**Published:** 2022-04-21

**Authors:** Xiaoxiao Sun, Linling Xu, Ying Xia, Shuoming Luo, Jian Lin, Yang Xiao, Gan Huang, Xia Li, Zhiguo Xie, Zhiguang Zhou

**Affiliations:** National Clinical Research Center for Metabolic Diseases, Key Laboratory of Diabetes Immunology (Central South University), Ministry of Education, and Department of Metabolism and Endocrinology, The Second Xiangya Hospital of Central South University, Changsha, China

**Keywords:** inflammasome, single nucleotide polymorphism, type 1 diabetes, correlation analysis, glutamic acid decarboxylase antibody

## Abstract

**Background and Aims:**

The NLRP3 gene is reportedly associated with several autoimmune diseases. However, in the Chinese Han population, whether NLRP3 polymorphisms are associated with type 1 diabetes (T1D) is unclear. Therefore, this study examined the associations of rs3806265 and rs4612666 of the NLRP3 gene with T1D susceptibility and the clinical characteristics of Chinese Han T1D patients.

**Methods:**

In total, 510 classic T1D patients and 531 healthy controls from the Chinese Han population were recruited for a case-control study. rs3806265 and rs4612666 of the NLRP3 gene were genotyped by MassARRAY. Logistic regression analysis and the chi-square test were used to compare the distributions of the alleles and genotypes of rs3806265 and rs4612666. The relationships between rs3806265 and rs4612666 and the clinical characteristics of T1D patients were analyzed by Kruskal-Wallis one-way ANOVA. Student’s t test was used to analyze normally distributed data. Bonferroni correction was used for multiple comparisons.

**Results:**

1) rs3806265 was associated with glutamic acid decarboxylase antibody (GADA) titers (P = 0.02), and patients with the CC genotype had higher GADA titers than patients with the TT genotype. 2) rs4612666 was also associated with GADA titers (P=0.041). Compared with patients with the CC genotype, patients with the TT genotype had higher GADA titers. 3) rs3806265 and rs4612666 of the NLRP3 gene were not significantly associated with T1D susceptibility under different genetic models.

**Conclusion:**

rs3806265 and rs4612666 of the NLRP3 gene were significantly associated with GADA titers in Chinese Han T1D patients.

## Introduction

The NLRP3 inflammasome is a kind of polyprotein complex composed of nucleotide-binding oligomerization domain-like receptor protein 3 (NLRP3), apoptosis-associated speck-like protein containing a caspase recruitment domain (ASC) and caspase-1 (1). The NLRP3 inflammasome can induce the maturation and secretion of pro-inflammatory factors such as IL-1β and IL18, leading to the inflammatory response (2). Because of the important role of the NLRP3 inflammasome, it is considered an effective target for the regulation of the occurrence and development of various kinds of autoimmune and inflammatory diseases (3).

T1D is an organ-specific autoimmune disease in which islet β cells are destroyed by autoreactive T cells, resulting in absolute insulin deficiency (4). On one hand, the loss of insulin results in excessive endogenous glucose production and a decrease in glucose uptake, thus causing hyperglycemia. On the other hand, the loss of insulin causes an increase in lipolysis, fatty acid oxidation and the excessive accumulation of ketones, leading to diabetic ketoacidosis.

Although the etiology of T1D is not yet fully understood, it is considered a complex inherited disease caused by genetic and environmental factors (5, 6). Genetic susceptibility is very important for the development of T1D (7–10). Studies have reported that in Caucasians, the risk of siblings all suffering from T1D is 6%, which is 15 times the risk in the general population (11). The consistency with which monozygotic twins are both affected by T1D (30–40%) is higher than that for dizygotic twins (6–8%) (11).

The NLRP3 gene, which is located on chromosome 1q44, is expressed in various types of cells, such as B cells, epithelial cells, and osteoblasts (12). The roles of the NLRP3 variants rs3806265 and rs4612666 in autoimmune and inflammatory diseases have been well researched. rs3806265 was reported to be associated with psoriatic juvenile idiopathic arthritis (13), recurrent aphthous stomatitis (14), inflammatory bowel disease (15) and so on, while rs4612666 was associated with periodontitis (16), food-induced anaphylaxis and aspirin-induced asthma (AIA) (17). Furthermore, rs4612666 is a functional variant that results in the enhanced stability of NLRP3 mRNA and an increase in the activity of NLRP3, which subsequently leads to a series of inflammatory responses (17).

Many studies have shown that the NLRP3 polymorphisms mentioned above are associated with several autoimmune diseases. With regard to T1D, it has been reported that there is an association between rs10754558 of the NLRP3 gene and T1D in the population of northeast Brazil (18). However, there have been no reports about whether the NLRP3 gene is correlated with T1D in the Chinese Han population. Given the roles played by rs3806265 and rs4612666 in autoimmune and inflammatory diseases and the fact that they have not been examined in relation to T1D in the Chinese Han population, we hypothesized that a relationship exists between these two SNPs and T1D in the Chinese Han population. In the present study, we further elucidated the etiology of T1D by investigating the associations of the two aforementioned SNPs in the NLRP3 gene with T1D in the Chinese Han population. This is the first report about the genetic association between NLRP3 polymorphisms (rs3806265 and rs4612666) and T1D.

## Materials and Methods

### Study Population

Every procedure of the study was approved by the Ethics Committee of Second Xiangya Hospital and followed the Helsinki Declaration guidelines. All participants signed written informed consent forms after the purpose and methods of the research were explained.

To evaluate the associations between the two SNPs and T1D as well as the clinical characteristics of T1D patients, a case-control study was performed, and 510 T1D patients (mean age 22 years; 275 males and 235 females) and 531 healthy people (mean age 42 years; 273 males and 258 females) were recruited from a Chinese Han population. The peak incidence of T1D appeared close to puberty; therefore, individuals recruited for the control group were much older, indicating that they had a lower likelihood of developing T1D in the future. The T1D patients were included when they were diagnosed in the Department of Metabolism and Endocrinology in the Second Xiangya Hospital. The recruitment criteria for T1D patients were as follows: (1) fulfilled the criteria for the diagnosis of diabetes developed by the WHO in 1999; (2) free from complications and did not receive additional medication except insulin injections; and (3) positive serum detection of one of the islet autoantibodies at diagnosis, such as GADA, IA-2A and ZnT8A (19, 20). In addition, patients with other kinds of diabetes, such as LADA and autoimmune diseases, and other kinds of autoimmune diseases were excluded through clinical examination. The healthy controls were recruited from an epidemiological study and physical examinations in the Second Xiangya Hospital and had normal clinical examination findings and no symptoms or a history of diabetes or autoimmune diseases. The inclusion criteria for the healthy controls were (1) a fasting plasma glucose (FPG) level < 5.6 mmol/l and (2) a 2-h postprandial plasma glucose (PPG) level < 7.8 mmol/l according to the 75 g OGTT test. To reduce the selection bias, we strictly followed the inclusion and exclusion criteria when enrolling the subjects.

### Clinical Measurements

Clinical measurements of the individuals were collected by well-trained investigators, including sex, weight, height, the age at T1D onset and some laboratory examination results, such as the FPG level, 2-h PPG level according to the OGTT test, fasting C-peptide (FCP) level and 2-h postprandial C-peptide (PCP) level. The FCP and 2-h PCP levels were detected by a chemiluminescence method (ADVIA Centaur XP Immunoassay System, Siemens, Germany) in the laboratory of the Second Xiangya Hospital, and the HbA1c level was detected by automated liquid chromatography (HLC-723G8, Tosoh, Japan). The radioligand binding assay was used in our laboratory to detect islet autoantibodies such as GADA, IA-2A and ZnT8A (21–23).

### DNA Extraction

A GeneNode Genomic DNA Extraction kit (Genenode Biotech Co., Ltd., Beijing) was used to extract DNA from peripheral whole blood samples in accordance with the manufacturer’s instructions. After extraction, the DNA samples were stored at -80°C.

### SNP Selection and Genotyping

We selected rs3806265 (chr1: 247423034(GRCh38.p12); T>A or T>C; Intron variant) and rs4612666 (chr1: 247435768 (GRCh38.p12); T>C; Intron variant) of the NLRP3 gene for analysis. These two SNPs were nominated using a widely used nomenclature system. We selected these two SNPs because they were previously demonstrated to be correlated with many autoimmune and inflammatory diseases, and rs4612666 was even shown to serve as a functional variant that influenced the process of inflammation. In addition, we prepared SNPs with minor allele frequencies (MAFs) >0.05 in Asian people. Those selected SNPs were located in different linkage regions.

MassARRAY was used for genotyping. ADS2.0 from Agena Bioscience was used to design the sequences of the primers for the two SNPs, which were then synthesized by BGI. Typer 4.0 software (Agena Bioscience Inc.) was used to collect genotyping data after clustering. A specificity of 0.90 and a sensitivity of 0.95 were set to cluster all loci. All genotyping assays were performed when the clinical information of the research subjects was unknown in the laboratory of BGI Genomics, BGI-Shenzhen, Shenzhen 518083, China.

### Statistical Analysis

The statistical analyses were performed with SPSS 20.0 software, and Hardy–Weinberg equilibrium (HWE) was detected by online software (http://ihg.gsf.de/cgi-bin/hw/hwa1.pl). We used the median (interquartile range (IQR)) to represent the values of continuous variables. The frequency distributions of the alleles and genotypes of the two SNPs between patients and controls were compared by the chi-square test and logistic regression. The clinical characteristics of patients with different genotypes of the two SNPs were compared by the chi-square test and the Kruskal-Wallis H test. Student’s t test was used to analyze the normally distributed data. P<0.05 was considered statistically significant. Bonferroni correction was used for multiple comparisons. The sample size was determined by Quanto software (version 1.2) with the following parameters: an expected odds ratio (OR) of 1.3, minor allele frequencies of 0.44 for rs3806265 and 0.44 for rs4612666 from an online website (http://pubs.broadinstitute.org/mammals/haploreg/haploreg.php), a prevalence of 1% of T1D patients in China and a statistical power of 0.8.

## Results

### Calculation of the Study Size by Using Quanto Software

The sample size calculated by Quanto software was 920, which is lower than the number of subjects who we actually recruited, suggesting that the sample size in our research was sufficient to detect an association between the two SNPs and T1D.

### Clinical and Biochemical Measurements of T1D Patients and Healthy Controls

The clinical and biochemical measurements of T1D patients and controls have been reported previously (24). There were differences between the cases and controls in many clinical characteristics. The sex distributions were not different between the cases and controls (275/235 vs. 273/258, p=0.418). T1D patients were younger (p<0.01) than the controls, and the FPG (p<0.001) and 2-h PPG (p<0.001) levels were both higher in the cases than in the controls.

### Detection of Hardy-Weinberg Equilibrium

The genotype frequencies of the two SNPs of the NLRP3 gene were in Hardy-Weinberg equilibrium (p>0.05), which means that the samples that we selected were representative. The results are shown in **Table 1**.

**Table 1:** Hardy-Weinberg equilibrium of NLRP3 gene polymorphisms.

Gene	SNP	Genotype	Observed value	Expected Value	χ^2^	P value
*NLRP3*	rs3806265	CC	132	127.80	0.533	0.465
		CT	257	265.41		
		TT	142	137.80		
*NLRP3*	rs4612666	TT	108	107.12	0.024	0.878
		TC	261	262.75		
		CC	162	161.12		

### Frequency Distributions of the Alleles and Genotypes of rs3806265 and rs4612666

The results are shown in **Table 2**. A total of 508 of the 510 cases of rs3806265 and 506 of the 510 cases of rs4612666 and 531 healthy controls were genotyped. No significant differences were found in the frequency distributions of the alleles and genotypes of the two SNPs in the cases and controls.

**Table 2:** Frequencies of the genotypes and alleles of rs3806265 and rs4612666 between cases and controls.

SNP	Cases n (%)	Controls n (%)	OR (95% CI)	P	Pc
rs3806265					
Genotype					
CC	118 (23.2)	132 (24.9)	1.007 (0.713-1.423)	0.966	1.000
CT	264 (52.0)	257 (48.4)	1.158 (0.862-1.555)	0.331	0.662
TT	126 (24.8)	142 (26.7)	1 (Reference)	–	
Allele					
C	500 (49.2)	521 (49.1)	1.006 (0.847-1.195)	0.944	1.000
T	516 (50.8)	541 (50.9)	–	–	
rs4612666					
Genotype					
TT	103 (20.4)	108 (20.3)	1.016 (0.717-1.441)	0.927	1.000
TC	251 (49.6)	261 (49.2)	1.025 (0.774-1.358)	0.864	1.000
CC	152 (30.0)	162 (30.5)	1 (Reference)	–	
Allele					
T	457 (45.2)	477 (44.9)	1.010 (0.849-1.201)	0.912	1.000
C	555 (54.8)	585 (55.1)	–	–	

SNP, single-nucleotide polymorphisms; OR, odds ratio; 95% CI, 95% confidence intervals. Pc, p value after Bonferroni correction.

### Associations Between the Two SNPs and Susceptibility to T1D Under Different Genetic Models

The results showed that rs3806265 and rs4612666 were not correlated with susceptibility to T1D under different genetic models. The results are shown in **Table 3**.

**Table 3:** The genetic models of rs3806265 and rs4612666 between cases and controls.

SNP	Minor allele	Genetic model											
		Dominant model			Recessive model			Overdominant model			Additive model		
		OR (95% CI)	P	Pc	OR (95% CI)	P	Pc	OR (95% CI)	P	Pc	OR (95% CI)	P	Pc
rs3806265	C	1.107 (0.838-1.462)	0.475	0.950	0.915 (0.688-1.216)	0.539	1.000	1.154 (0.904-1.471)	0.250	1.000	1.006 (0.847-1.195)	0.944	1.000
rs4612666	T	1.022 (0.784-1.333)	0.870	1.000	1.001 (0.740-1.355)	0.995	1.000	1.018 (0.798-1.299)	0.884	1.000	1.010 (0.850-1.200)	0.912	1.000

SNP, single-nucleotide polymorphisms; OR, odds ratio; 95% CI, 95% confidence intervals. Pc, p value after Bonferroni correction.

### Associations Between the Two SNPs and the Clinical Characteristics of T1D Patients

We performed a genotype-phenotype association analysis combining basic information such as age, sex and duration of the disease and biochemical measurements such as HbA1c and FCP level and the titer and positivity rate of islet autoantibodies with different genotypes of the two SNPs. The data are shown in **Tables 4**, **5**. rs3806265 was significantly associated with the titer of GADA (P = 0.02), and patients with the CC genotype had higher GADA titers than patients with the TT genotype. rs4612666 was also associated with the GADA titer (P=0.041). Compared with patients with the CC genotype, patients with the TT genotype had higher GADA titers.

**Table 4:** Clinical and biochemical characteristics of T1D patients with different genotypes of rs3806265 of the NLRP3 gene.

Clinical characteristics	Genotype			P value
	CC	CT	TT	
Sample size	118	264	126	–
Sex (men/women)	74/44	135/129	65/61	0.092
Onset age (year)	22 (11-35)	18 (11-32)	19 (11-28)	0.496
T1D duration (months)	4.75 (0.72-18.75)	5.50 (0.41-20.25)	3.50 (0.36-24.00)	0.877
BMI (kg/m^2^)	19.17 (16.80-21.00)	18.74 (16.38-20.60)	18.44 (16.50-20.80)	0.534
FCP (pmol/L)	74.78 (14.75-152.49)	77.50 (19.72-169.00)	74.38 (13.49-227.24)	0.615
PCP (pmol/L)	142.90 (36.84-277.90)	141.10 (47.22-276.22)	129.34 (23.73-265.22)	0.714
HbA1c (%)	9.70 (7.95-12.25)	10.10 (7.70-12.88)	10.30 (7.40-12.80)	0.821
GADA positive (%)	87.29%	87.07%	92.06%	0.327
GADA titer (U/mL)	498.59 (105.88-917.22)	334.36 (89.98-721.02)	220.44 (68.63-662.79)	0.020*
IA-2A positive (%)	42.06%	47.93%	49.14%	0.510
IA-2A titer (U/mL)	202.80 (59.39-636.90)	190.66 (44.98-665.02)	191.02 (56.17-641.96)	0.988
ZnT8A positive (%)	28.42%	29.60%	35.71%	0.469
TG (mmol/L)	0.94 (0.71-1.49)	1.00 (0.71-1.49)	0.90 (0.63-1.17)	0.247
TC (mmol/L)	4.23 (3.56-4.83)	4.34 (3.75-5.11)	4.04 (3.43-4.79)	0.217

SNP, single-nucleotide polymorphisms; BMI, body mass index; FCP, fasting C-peptide; PCP, 2-h postprandial C-peptide; HbA1c, glycated hemoglobin; GADA, glutamic acid decarboxylase antibody; IA-2A, protein tyrosine phosphatase antibody; ZnT8A, zinc transporter 8 antibody. The symbol "*" means the difference was statistically significant.

**Table 5:** Clinical and biochemical characteristics of T1D patients with different genotypes of rs4612666 of the NLRP3 gene.

Clinical characteristics	Genotype			P value
	TT	TC	CC	
Sample size	103	251	152	–
Sex (men/women)	66/37	128/123	79/73	0.068
Onset age (years)	21 (11-35)	18 (11-32)	20 (11-30)	0.616
T1D duration (months)	3.00 (0.60-18.00)	5.00 (0.50-16.00)	5.00 (0.37-36.00)	0.693
BMI (kg/m^2^)	19.17 (16.90-21.01)	18.64 (16.00-20.59)	18.60 (16.69-20.81)	0.285
FCP (pmol/L)	79.00 (15.36-156.07)	73.03 (19.13-164.28)	74.38 (14.61-163.52)	0.986
PCP (pmol/L)	142.90 (36.46-278.64)	140.91 (46.65-273.80)	130.48 (26.34-267.79)	0.739
HbA1c (%)	9.70 (7.90-12.65)	10.10 (8.00-12.90)	9.85 (7.28-12.50)	0.745
GADA positive (%)	87.40%	88.00%	89.40%	0.870
GADA titer (U/mL)	439.00 (113.70-900.79)	320.00 (79.34-775.74)	254.14 (76.11-677.11)	0.041*
IA-2A positive (%)	35.50%	48.70%	50.00%	0.059
IA-2A titer (U/mL)	327.55 (78.59-734.33)	167.47 (41.97-659.52)	210.94 (57.17-658.83)	0.257
ZnT8A positive (%)	27.70%	29.70%	35.80%	0.389
TG (mmol/L)	0.93 (0.68-1.46)	0.98 (0.70-1.57)	0.94 (0.68-1.21)	0.521
TC (mmol/L)	4.23 (3.53-4.84)	4.33 (3.76-4.83)	4.13 (3.63-5.17)	0.814

SNP, single-nucleotide polymorphisms; BMI, body mass index; FCP, fasting C-peptide; PCP, 2-h postprandial C-peptide; HbA1c, glycated hemoglobin; GADA, glutamic acid decarboxylase antibody; IA-2A, protein tyrosine phosphatase antibody; ZnT8A, zinc transporter 8 antibody. The symbol "*" means the difference was statistically significant.

## Discussion

NLRP3, a key protein in the NLRP3 inflammasome, is essential for innate and acquired immunity. Therefore, some authors have proposed that NLRP3 plays a crucial role in the maintenance of tolerance conferring protection from autoimmune diseases (25, 26). NLRP3 mutations have been reported to lead to many autoimmune disorders (27, 28), but only a few disease-causing variants are associated with T1D (18).

T1D is an autoimmune disease characterized by impaired β cell function caused by autoreactive T cells. The positivity rate for the detection of islet autoantibodies in T1D patients is significantly higher than the rates in patients with other kinds of diabetes and healthy people. GADA, one of the main types of islet autoantibodies in T1D, is a marker of immunity in the early stage of the disease. Furthermore, a high titer of GADA strongly predicts the development of thyroid autoimmunity in T1D, suggesting that an association exists between a high titer of GADA and severe autoimmunity. Several studies also pointed out that a high titer of GADA was associated with progressive β cell failure in patients with slowly progressing T1D (29).

To date, this study is the first to clarify the associations between these two SNPs (rs3806265 and rs4612666) of the NLRP3 gene and T1D in a Chinese Han population. No association of the NLRP3 gene with susceptibility to T1D was found. However, significant differences were found between the two SNPs of the NLRP3 gene with regard to the GADA titer in T1D patients. Due to the limited number of our samples, further validation in a larger population is required as well as in other countries and ethnic groups. The lack of associations between the selected SNPs and susceptibility to T1D might be partly explained by the fact that two SNPs may not be fully representative of the entire gene. In addition, T1D is a multigenic disease; therefore, the effect of a single gene might be small. Furthermore, as a multifactorial disease, T1D is also caused by environmental factors. In future studies, we may consider involving larger sample sizes and more SNPs of the NLRP3 gene. Regarding the associations between the two selected SNPs and clinical characteristics of patients with T1D, both rs3806265 and rs4612666 of the NLRP3 gene were associated with the GADA titer. T1D patients with the CC genotype of rs3806265 had a higher GADA titer than patients with the TT genotype, while patients with the TT genotype of rs4612666 had a higher GADA titer than those with the CC genotype. Patients with different genotypes of the two SNPs had different GADA titers, which may indicate that residual β cell function and disease progression would also be different. We speculate that the two SNPs may influence the activation of the NLRP3 inflammasome therefore the process of cytokines factors production by NLRP3 inflammasome. The cytokines such as IL-18 and IL-1β, can enhance T and B lymphocyte responses and may play a role in the production of GADA. Functional experiments will be needed to explore the mechanism by which rs3806265 and rs4612666 affect the GADA titers of T1D patients. The GADA titer reflects the severity of cellular immune destruction and can predict the risk of β cell failure as well as the progression of T1D (30). The correlation we found in this study between the two SNPs and the GADA titer can be applied to the clinical treatment and prognosis of T1D in the future. Studies from our group have shown that GADA titers are associated with the loss of β-cell function (31), the rate of the loss of β-cell function in LADA patients with high GADA titers was significantly faster than in patients with low GADA titers. Early insulin therapy can delay the apoptosis of βcell and protect the function of the remaining βcells (32). In this research we found there was a relationship between the two SNPs of NLRP3 gene and GADA titers, therefore we suggest early insulin therapy for patients carrying the genotype associated with a high titer of GADA. Besides, studies from our group have shown that high titers of GADA is a strong predictor of the development of thyroid autoimmunity in patients with type 1 diabetes and latent autoimmune diabetes in adults (33), therefore clinicians should also focus more on islet function and glycemic control as well as the appearance of other autoimmune diseases in patients carrying the genotype associated with high titers of GADA.

In conclusion, this case-control study including 510 cases and 531 controls demonstrated that there are associations between rs3806265 and rs4612666 of the NLRP3 gene and the GADA titers in T1D patients. These findings have deepened our understanding of the pathophysiology of T1D, and the differences in the GADA titers in patients with different genotypes of the two SNPs will help us predict the residual β cell function as well as disease progression of T1D patients, thus providing new directions for disease prevention and treatment in the future.

## Data Availability Statement

The original contributions presented in the study are included in the article/supplementary material. Further inquiries can be directed to the corresponding authors.

## Ethics Statement

The studies involving human participants were reviewed and approved by the Ethics Committee of Second Xiangya Hospital. Written informed consent to participate in this study was provided by the participants’ legal guardian/next of kin.

## Author Contributions

Conception and design: ZX. Administrative support: ZZ. Provision of study materials or patients: ZZ, ZX, XL, YaX, GH, XS, JL, SL, and YiX. Collection and assembly of data: ZX, XS, and LX. Data analysis and interpretation: ZX, XS, and LX. Manuscript writing: LX and XS. Final approval of manuscript: All authors. All authors are in agreement with the content and the submission of the manuscript.

## Funding

This work was supported by the National Natural Science Foundation of China (82070813,81873634, 81400783), the National Key R&D Program of China (2016YFC1305000, 2016YFC1305001, 2018YFC1315603), the Science and Technology Major Project of Hunan Province (2017SK1020), and the Hunan Natural Science Funds for Distinguished Young Scholars (Grant No.2020JJ2053).

## Conflict of Interest

The authors declare that the research was conducted in the absence of any commercial or financial relationships that could be construed as a potential conflict of interest.

## Publisher’s Note

All claims expressed in this article are solely those of the authors and do not necessarily represent those of their affiliated organizations, or those of the publisher, the editors and the reviewers. Any product that may be evaluated in this article, or claim that may be made by its manufacturer, is not guaranteed or endorsed by the publisher.
